# Histological and immunohistochemical characterization of granulomas in alpacas (*Vicugna pacos*) naturally infected with tuberculosis

**DOI:** 10.3389/fvets.2025.1638459

**Published:** 2025-09-24

**Authors:** Irene Agulló-Ros, Inés Ruedas-Torres, Laura Hunter, Alison Bird, Claire E. Whitehead, Francisco J. Salguero

**Affiliations:** ^1^Department of Anatomy and Comparative Pathological Anatomy and Toxicology, Research Group GISAZ, UIC Zoonosis and Emerging Diseases ENZOEM, University of Córdoba, Córdoba, Spain; ^2^Pathology, UK Health Security Agency (UKHSA), Porton Down, Salisbury, United Kingdom; ^3^Faculty of Health and Medical Sciences, University of Surrey, Guildford, United Kingdom; ^4^Camelid Veterinary Services Ltd, Reading, United Kingdom

**Keywords:** tuberculosis, alpacas, granuloma, immunohistochemistry, cell marker, *Mycobacterium tuberculosis* complex

## Abstract

Tuberculosis (TB), caused by the *Mycobacterium tuberculosis* complex (MTBC), is a chronic zoonotic disease of increasing concern in alpacas (*Vicugna pacos*), a species highly susceptible to the disease. Given the growing alpaca population in Europe and zoonotic potential, understanding TB pathology in alpacas is crucial. This study provides the first comprehensive histopathological and immunohistochemical characterization of TB lesions in naturally infected alpacas. Granulomas from the lungs (*n* = 175), liver (*n* = 241), and lymph nodes (*n* = 55), were classified into four developmental stages (I, II, III and IV) based on their morphology, necrosis, fibrosis, cellular composition, and presence of acid-fast bacilli (AFBs). Advanced granulomas (stages III and IV) predominated in all tissues, indicating chronic infection. High numbers of AFBs were observed in lung and lymph node granulomas across all stages, with very rare presence of multinucleated giant cells (MNGCs). This pattern in the lung, with extensive necrosis and lack of fibrous encapsulation, together with the presence of abundant AFBs, suggests deficient immune control and significant transmission risk. In contrast, liver granulomas, particularly encapsulated stage IV lesions, showed fewer detectable AFBs, implying better mycobacterial control in this organ. Immunohistochemistry in selected granulomas revealed ionized calcium-binding adaptor protecin (IBA1) immunopositive macrophages were most prevalent cells in early stages (stage I and II), while T (CD3+) and B lymphocytes (B-cell specific activator protecin+) increased in advanced (stage III and IV) granulomas, forming peripheral lymphoid follicle-like structures. Neutrophils (immunopositive to myeloperoxidase) were less abundant, but more prominent in advanced lesions showing extensive necrosis. The high incidence of liver lesions suggests high dissemination of pathogenic mycobacteria and generalized tuberculosis in this species. This research fills knowledge gaps about tuberculous granulomas in camelids and highlights alpacas as potential sources of mycobacterial excretion, posing a transmission risk to domestic animals, wildlife, and humans.

## 1 Introduction

Tuberculosis (TB) is a chronic, zoonotic and granulomatous disease caused by members of the *Mycobacterium tuberculosis* complex (MTBC) (1–3). It emerges as one of the most important and widespread infectious diseases, representing the world’s deadliest bacterial infection and contributing to substantial global economic losses (4). TB affects a broad range of domestic (1, 5) and wild mammal species (6–13), including South American Camelids (SACs) (14–25). Notably, llamas (*Lama glama*) and alpacas (*Vicugna pacos*) are considered highly susceptible to TB induced by both *Mycobacterium bovis* and *Mycobacterium microti* (26–28), which has been reported in other animal species (29–31), and humans (32).

Alpacas have gained increasing importance in recent decades. Originally bred for their high-quality wool, they are now also employed in agritourism, recreation, and even in pet-therapies. The United Kingdom is home to the largest alpaca population in Europe, reaching 60,000 individuals in February 2023, according to The British Alpaca Society (http://www.bas-uk.com/) (33, 34). However, alpaca numbers have also significantly increased in other European countries, underscoring the importance of this species (15, 18).

In the last decades, TB outbreaks in alpacas have heightened, primarily associated with areas exhibiting a high bovine TB prevalence in wildlife and domestic species, especially cattle (10, 15, 17, 35). Mycobacterial infection might go undetected until the first deaths occur in the herd, preceded by nonspecific clinical signs such as depression, weight loss, lethargy, anorexia, and respiratory distress (20). While respiratory clinical signs are commonly associated with extensive respiratory pathology, they may not always be apparent, even in the presence of severe lung lesions, thereby facilitating the spread of pathogenic mycobacteria within the herd before succumbing to the disease (36). Macroscopic findings consist of multifocal to coalescing foci of granulomatous inflammation of different size, with caseotic necrosis, and observed in several organs, including the lungs, liver and lymph nodes (LNs) (15, 18, 36). The lungs and LNs usually show a diffuse granulomatous inflammation pattern, indicative of “open TB,” whereas the presentation in other organs, is often characterized by well-delimited granulomas (15).

Due to the high susceptibility to the disease and the reported cases of “open TB” in alpacas, it has been suggested that this species could act as a reservoir for MTBC (15), becoming a source of infection not only for wild and domestic animals, but also for humans (15, 17). Notably, there are two documented cases of human *M. bovis* infection following contact with an infected alpaca; a case of cutaneous TB in a veterinary surgeon following post-mortem examination (3) and a case of pulmonary TB in an alpaca owner (37). These cases highlight the zoonotic risk associated with alpacas, a species in close contact with humans. Comprehensive studies on the histopathological aspects of granulomatous lesions in alpacas infected with MTBC members are essential for a detailed understanding of the disease’s manifestation in this species.

In this study, we used samples from naturally MTBC-infected alpacas to characterize the post-mortem lesions microscopically and categorize granulomas according to a scoring system. Additionally, a panel of commercially available antibodies was employed to study the presence and distribution of several cell populations within the granulomas.

## 2 Materials and methods

### 2.1 Case study

The study farm comprised approximately 700 alpacas at the start of a TB breakdown. The farm was in Oxfordshire within the area classified by DEFRA as Edge Area with regard to risk for bovine TB. A representative proportion of the herd according to the statistical package approved by DEFRA had undergone Enferplex testing in June 2018 with a negative herd test. The alpacas were kept primarily out at pasture although the weanling group and a small number of underweight alpacas were housed at night in a well-ventilated barn. The farm was using 5 different locations within a 2-mile radius. At the start of the breakdown approximately 170 non-pregnant adult female alpacas were being kept on rented land within the same radius of land to rest pasture on the main farm. Some cattle had been grazed on an orchard adjacent to this land for a period of 6 weeks during the previous summer; the cattle had originated from a farm that had been a TB breakdown herd until recently prior to being moved to that orchard. The first clinical case of TB occurred in January 2019 in an alpaca on that rented land: it was moved to the main farm, to a sick pen in the main barn and was evaluated by the regular farm veterinarian. This alpaca was being treated for pneumonia for 10 days before it died. A post-mortem examination (PME) was performed on farm at which TB-like lesions were found in the lungs, liver, spleen and various lymph nodes. Six further clinical cases occurred from the same group over the next 4 months: one alpaca died while five were euthanased. PME was performed in 4 out of the 6 six cases and TB-like lesions were found.

Skin testing was performed on alpacas in the affected group (*n* = 166). Ten skin test reactors were culled, 8 of which had no visible lesions at PME, while one reactor had TB-like lesions in the pleura and mediastinal lymph nodes.

One month after the first death, a weanling that had been housed in the barn adjacent to the index case was found dead and found to have TB-like lesions at PME. The remaining alpacas on the farm underwent skin (*n* = 428) and serological (*n* = 563, including the original group) testing and reactors were culled. All initial skin test reactors were subjected to PME. The farm remained under restriction for a total of 4 years, undergoing 5 rounds of statutory parallel skin and serological testing until September 2020, after which only skin testing was performed, supplemented by privately funded serological testing.

PMEs were performed on a total of 233 alpacas during the course of the breakdown. Samples were collected from affected organs with visible lesions, immersed in 10% neutral buffered formalin (NBF) and stored until processing. Samples were also submitted for culture from 49 alpacas during the breakdown.

### 2.2 Bacteriology/pathogen detection

Samples from the original case cultured positive for MTBC while another clinical case from that group cultured positive for *Mycobacterium bovis.* Out of 10 skin test reactors from the initial affected group, one alpaca had lesions in the pleura and mediastinal lymph nodes and cultured positive for *M. bovis* while 8 had no visible lesions (NVL). Three of these NVL animals cultured positive for *M. vaccae* (*n* = 2) or *M. terrae* (*n* = 1). The first weanling that died from the main farm cultured positive for *M. bovis.* Eight out of nine of the weanling group that were submitted cultured positive for *M. bovis.* Further samples were submitted for culture to the Regional Centre for Mycobacteriology in Cardiff from selected individuals (*n* = 27). Some cultured positive for *M. bovis* (35%), others for MTBC (37%), or were negative (28%).

The index case was submitted for PCR testing at the University of Liverpool Schol of Veterinary Medicine, and this was positive for MTBC but the laboratory was unable to complete speciation on this sample.

### 2.3 Histopathology

Selected samples from lung (*n* = 12), liver (*n* = 40), and lymph node (retropharyngeal, submandibular and mediastinal) (*n* = 10) exhibiting TB-like lesions were fixed by immersion in 10% NBF (Solmedia Ltd., Shrewsbury, United Kingdom) and processed using routine histological methods into paraffin blocks. All tissue blocks were sectioned at 4 μm and stained with haematoxylin and eosin (H&E) for histological evaluation.

Stained slides were scanned either with a Hamamatsu S360 (Hamamatsu Photonics, Shizuoka, Japan) or a 3D-Histech (3DHISTECH Ltd., Hungary) digital scanner and e-slides were evaluated using CaseViewer software (v 2.4.0.119028) (3DHISTECH Ltd., Hungary) and ndp.view2 software (v 2.9.29) (Hamamatsu Phonics, Japan).

Consecutive sections were stained with the Ziehl-Neelsen (ZN) technique to detect acid-fast bacilli (AFBs). The quantification of AFBs was performed through light microscopy on ZN-stained slides, following the methodology previously described by Garcia-Jimenez et al. (2012) (8). Briefly, the total count of AFBs present in each granuloma was determined and recorded using a scoring system as follows: 0 = no AFBs, 1 = 1–10 AFBs, 2 = 11–50 AFBs; and 3 ≥ 50 AFBs.

In areas exhibiting focal extensive pyogranulomatous lesions, supplementary Gram staining was performed for the identification of non-mycobacterial bacterial colonies by light microscopy.

### 2.4 Immunohistochemistry

For the immunohistochemical (IHC) study, 12 lung and 22 liver samples were selected based on the presence of representative tuberculous granulomas from each developmental stage as selection criteria, after analyzing the H&E stained sections. The analysis included a representative number of granulomas per stage to ensure robust evaluation. Given the fact that granulomas do not consistently appear in the same tissue plane, the number of granulomas evaluated varied between sections and IHC markers. In lung tissue sections, the average number of granulomas examined per stage and IHC marker was: stage I (29.5 ± 24.1), stage II (26.3 ± 12.6), stage III (68.5 ± 19.1), and stage IV (21.5 ± 8.2). In liver tissue sections, the corresponding counts were: stage I (24.0 ± 15.0), stage II (27.0 ± 15.3), stage III (28.3 ± 5.4), and stage IV (50.0 ± 11.7). Each IHC marker was applied to independent serial sections of the same tissue samples to assess specific cell populations. IHC staining was performed to study different cell populations within the lung and liver granulomas, including macrophages (IBA1+), neutrophils (myeloperoxidase; MPO+), B-cells (B-cell Specific Activator Protein, SAP+; Pax-5) and T cells (CD3+). Additionally, the presence of fibrous tissue was demonstrated on representative tissue sections containing lesions by using an anti-vimentin antibody. Details of primary antibodies and IHC methods are summarized in Table 1. Briefly, for all cases, deparaffinization and heat-induced epitope retrieval (HIER) for 20 min at 95 °C were performed on a Leica BOND-RXm. HIER was achieved using BOND Epitope Retrieval Solution 1 (ER1, pH 6.0) for Vimentin, IBA1, MPO and CD3 antibodies, whereas as for B-cell-SAP, the BOND ER2 (pH 3.2) was used. Following primary antibody incubation, immunostaining was performed with the Dako Real EnVision Detection System Peroxidase/DAB, Rabbit/Mouse (Agilent, CA, USA) and counterstained with Gill’s haematoxylin. Subsequently, slides underwent routine dehydration and were mounted using the Ecomount medium (Biocare Medical, CA, USA). To ensure specificity, negative controls, involving the replacement of primary Ab with a blocking solution (OMIT), and isotype controls were included in each run.

**Table 1:** Summary of immunohistochemical methods: primary antibody details, source, dilution and blocking solution.

Specificity/Clone	Type of antibody	Dilution	Blocking solution	HIER	Source
Vimentin	mAb (clone, V9)	1:100^1^	Superblock^2^	ER1^3^	Agilent, CA, USA
IBA1	mAb (clone, GT10312)	1:100^1^	Superblock^2^	ER1^3^	Invitrogen, MA, USA
MPO	pAb	1:100^1^	Superblock^2^	ER1^3^	Invitrogen, MA, USA
CD3	pAb	1:200^1^	Superblock^2^	ER1^3^	Agilent, CA, USA
B-Cell-SAP	mAb (clone, DAK-Pax5)	1:25^1^	Superblock^2^	ER2^4^	Agilent, CA, USA

^1^Primary antibody incubation 1 h in the oven at 37^o^ C; ^2^Superblock (TBS) Blocking Buffer (Thermo Scientific, USA); ^3^BOND Epitope Retrieval Solution 1 (pH 6.0); ^4^BOND Epitope Retrieval Solution 2 (pH 9); HIER, heat-induced epitope retrieval; MAb, monoclonal antibody; pAb, polyclonal antibody; MPO, myeloperoxidase; B-cell SAP, B-cell Specific Activator Protein.

Additionally, IHC-stained slides were scanned using a high-resolution digital slide scanner, and digital image analysis was performed with Nikon NIS-Elements AR software (Nikon Instruments Inc., NY, USA). For each slide, individual granulomas were manually drawn as regions of interest (ROIs) based on clear histopathological identification. In all granulomas, the necrotic core was excluded from the analysis, except for the MPO staining, in which the entire lesion was analysed. Within each ROI, automated color deconvolution was applied to separate the positive immunolabeling from the hematoxylin counterstain. A fixed threshold intensity was defined for each marker based on control tissues and applied uniformly across samples to ensure consistency. The software then calculated the immunolabeled area as a percentage of the total ROI area. Image analysis was performed once, and both the parameter configuration and the resulting data were jointly examined by two veterinary pathologists to ensure analytical consistency and data integrity.

Histopathological and immunohistochemical analyses were conducted in a laboratory compliant with ISO 9001:2015 and Good Laboratory Practice (GLP) standards.

### 2.5 Statistical analysis

Figures and data analyses were performed using GraphPad Prism 10.2.3 software (GraphPad Prism Inc., San Diego, CA, United States). Non-parametric ANOVA (Kruskall-Wallis) followed by multiple comparison test was used to compare median values of positive immunostaining for each marker and granuloma stage. *P*-value below 0.05 was considered indicative of statistical significance, indicated with *(*p* ≤ 0.05), **(*p* ≤ 0.01), and ***(*p* ≤ 0.001).

## 3 Results

### 3.1 Scoring of granulomas (I-IV)

The tuberculous granulomas in the lungs, liver and LN were classified microscopically into 4 stages of development according to their size, abundance and spatial distribution of inflammatory cellular elements, presence of AFBs, extent of necrosis and mineralization, as well as the presence of fibrosis (Table 2). Lesions were located randomly within the lung, liver and LN parenchyma. A description of the morphological characteristics of each developmental stage is provided below:

**Table 2:** Number and type of tuberculous granulomas in alpacas.

Tissue	Granuloma stage				Total count
	I	II	III	IV	
Lung	18	37	83	37	175
Liver	43	54	88	56	241
Lymph nodes	0	0	34	21	55

Total number of granulomas observed at each stage in lungs, liver and lymph nodes.

**Figure 1:**
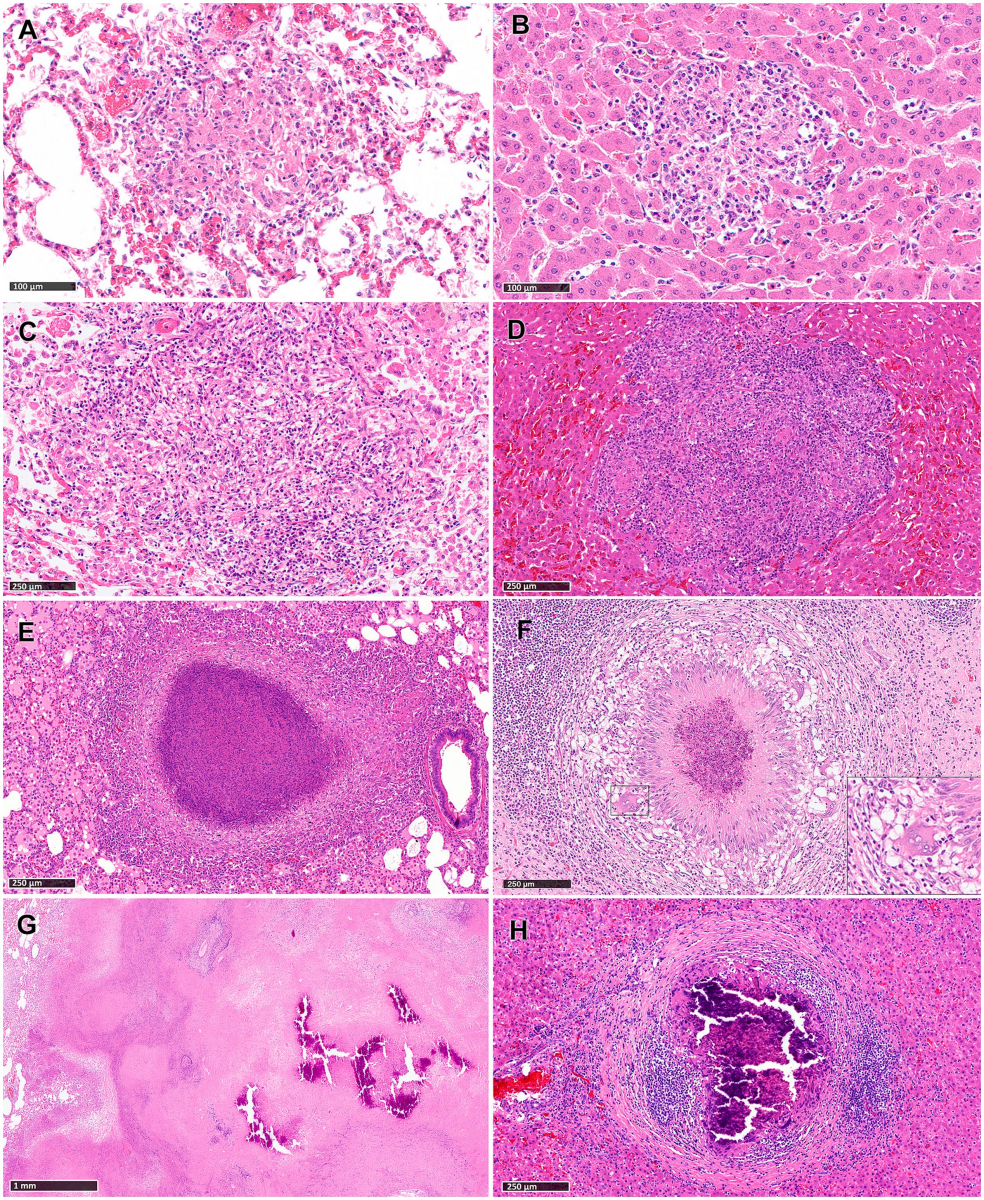
Tuberculous granulomas, alpacas, hameotoxilin and eosin (H&E). **(A,B)** Stage I granulomas in lung **(A)** and liver **(B)**. Small and unencapsulated structures composed primarily by macrophages and interspersed lymphocytes. Stage II granulomas in lung **(C)** and liver **(D)**. Organized granulomas with epithelioid macrophages and lymphocytes with foamy macrophages and neutrophils interspersed. Stage III granulomas in lung **(E)** and liver (**F**; insert showing a MNGC). Organised, with central caseous necrosis with degenerated neutrophils, surrounded by a rim of epithelioid macrophages. Stage IV granulomas in lung **(G)** and liver **(H)**. **(G)** Advanced tuberculous lesion featuring extensive and irregular central caseous necrotic and mineralized areas. **(H)** Highly organized granuloma exhibits prominent caseous necrosis with mineralization and surrounded by connective tissue and dense clusters of lymphocytes. Scale bars **(A,B)** = 100 μm. Scale bars **(C–F,H)** = 250 μm. Scale bar **(G)** = 1 mm.

Stage I: these lesions comprised small, unencapsulated and well demarcated structures. They consisted primarily of clusters of macrophages and interspersed lymphocytes, with occasional, scattered foamy cells and neutrophils. Figures 1A,B show representative Stage I granuloma in lung and liver, respectively. In some stage I granulomas, epithelioid macrophages were also observed, while Langhan’s type multinucleated giant cells (MNGCs) were very rare.

Stage II: these lesions exhibited larger dimensions compared to those in Stage I, and were unencapsulated, circumscribed, with well-demarcated borders and round morphology. Granulomas at this stage were composed primarily of scattered macrophages, often epithelioid, and lymphocytes, alongside variable numbers of foamy macrophages and neutrophils. Minimal necrotic areas were sometimes present in the center of the lesions, generally comprising necrotic inflammatory cells. Occasional MNGCs were also observed. Figures 1C,D show representative Stage II granuloma in lung and liver, respectively.

Stage III: granulomas at this stage were characterized by central necrosis, composed of numerous degenerate neutrophils along with nuclear pyknosis and karyorrhexis. Varying amounts of early caseous necrosis were also observed, primarily consisting of homogeneous, eosinophilic material. Epithelioid and foamy macrophages with very rare MNGCs, encircled the necrotic areas. The outermost periphery was comprised of macrophages, lymphocytes, plasma cells, and neutrophils, sometimes surrounded by a connective tissue capsule (Figures 1E,F).

Stage IV: these lesions represented a variable morphology, from round to any shape, that included irregular, large, multifocal granulomas partially encapsulated, and highly organized granulomas with a thick fibrotic capsule. Multifocal granulomas presented extensive and irregular central necrotic areas, which were caseous and mineralized, surrounded by an incomplete connective tissue capsule (Figure 1G). Epithelioid and foamy macrophages with occasional MNGCs encircled the necrotic areas, with lymphocytes and interspersed neutrophils observed in the periphery. Highly organized granulomas were characterized by a prominent caseous necrosis, often associated with concomitant, dystrophic mineralization, surrounded by thick connective tissue (Figure 1H). The necrotic core was encircled by a rim of epithelioid and foamy macrophages together with very few MNGCs, with notably dense clusters of lymphocytes near the peripheral capsule (Figure 1H).

**Figure 2:**
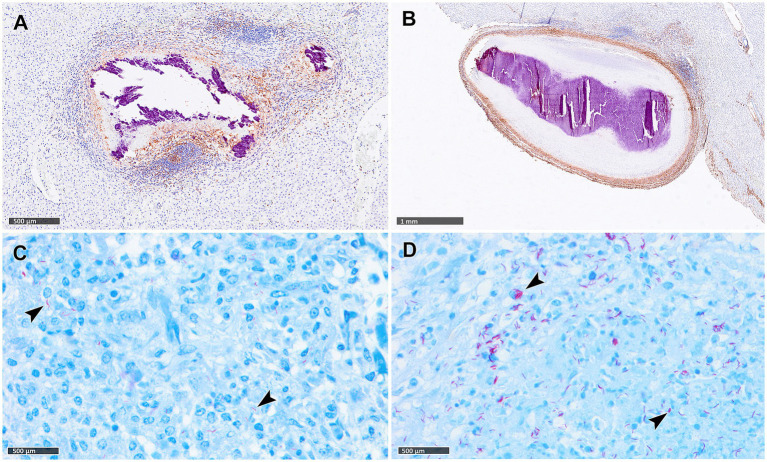
Tuberculous granulomas, alpacas, vimentin immunohistochemical detection and Ziehl-Neelsen (ZN) staining. **(A)** Stage IV granuloma with the liver parenchyma showing weak fibrotic rim (IHC, vimentin). **(B)** Stage IV granuloma within the liver parenchyma showing a thick vimentin immunopositive fibrotic capsule surrounding the lesion. **(C,D)** Acid-fast bacilli (AFBs, black arrowheads) in lungs of alpacas with stage II **(C)** and stage IV **(D)** tuberculous granuloma (ZN staining). Scale bar **(A)** = 1 mm. Scale bars **(B–D)** = 500 μm.

Reactive fibroplasia, leading to the development of a fibrous capsule, was noted in the liver tissue samples as confirmed by vimentin immunolabelling (Figures 2A,B). Occasionally, in some granulomas that developed adjacent to Glisson’s capsule, pre-existing connective tissue was incorporated into the granuloma structure (Figure 2B). Gram staining revealed no evidence of non-mycobacterial bacteria in the diffuse granulomatous inflammation observed in some lung, liver and LN samples.

### 3.2 Granuloma development and acid-fast bacilli distribution in different organs: a comparative analysis

The total number of granulomas at each developmental stage in lung, liver and LNs is summarized in Table 2. Advanced granulomas (stages III and IV) predominated in all the evaluated tissues compared to early granulomas (stage I and II). Moreover, the LN with TB-like lesions included in the study exhibited exclusively advanced granulomas.

The number of granulomas showing AFB scores ranging from 0 to 3, together with the presence and frequency of AFBs in each granuloma stage per tissue are summarized in Table 3. In the lung, a correlation was noted between the progression of lesion development and an increase in the total number of AFBs observed in granulomas. AFBs were detected in stage I, II (Figure 2C), III and IV (Figure 2D) granulomas, with a tendency for higher numbers of AFBs visible in stage IV (Figure 2D). Notably, almost all stage IV granulomas contained more than 50 AFBs (Table 2). Conversely, liver samples exhibited lower counts of AFBs, with early stages I and II granulomas showing higher numbers of AFBs than stages III and IV. In LNs, AFBs were also observed in advanced granulomas, with the presence of mycobacteria consistently detected in stage IV.

**Table 3:** Tuberculous granulomas, alpacas.

Granuloma stage	Lung					Liver					LNs				
	0	1	2	3	Total score	0	1	2	3	Total score	0	1	2	3	Total score
Stage I	10	4	3	1	0.72	11	32	0	0	0.74	0	0	0	0	–
Stage II	2	14	9	12	1.84	11	35	6	2	0.98	0	0	0	0	–
Stage III	2	26	4	51	2.25	65	13	7	3	0.40	6	13	2	13	1.65
Stage IV	0	1	4	32	2.85	50	3	1	2	0.19	0	9	3	9	2

Number of granulomas displaying acid fast bacilli (AFB) scores ranging from 0 to 3 and the total score (mean) for the quantity of AFBs in granulomas at each stage in the lung, liver, and lymph nodes (LNs). The total number of AFBs present in each granuloma were counted and recorded using a scoring system: 0 = no AFBs, 1 = 1–10 AFBs, 2 = 11–50 AFBs; and 3 ≥ 50 AFBs. AFB, acid fast bacilli; LNs, lymph nodes.

### 3.3 Distribution of cell populations within granulomas

**Figure 3:**
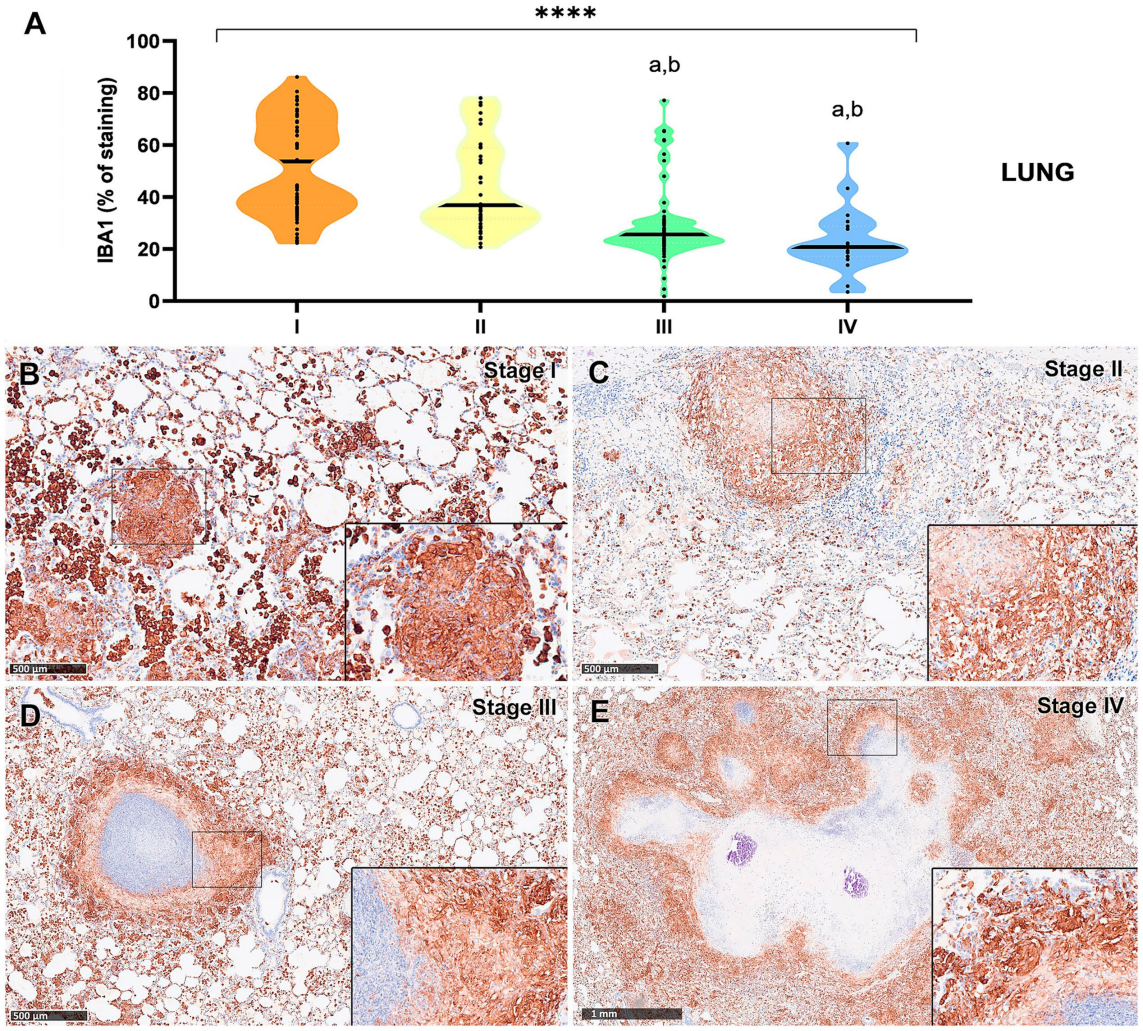
Tuberculous granulomas, alpacas, IBA1 immunolabelling in stage I, II, III and IV granulomas in the lung. **(A)** Percentage of IBA-immunopositive staining in stage I to IV granulomas. **(B)** IBA1 expression in stage I granuloma. **(C)** IBA1 expression in stage II granuloma. **(D)** IBA1 expression in stage III granuloma surrounding a necrotic core. **(E)** IBA1 positive labelling surrounding a necrotic core in stage IV granuloma. Insets show close-up images at higher magnification showing IBA1-immunopositive macrophages. Scale bars **(B–D)** = 500 μm. Scale bar **(E)** = 1 mm (insets = 100 μm). *p < 0.05; ***p < 0.01; ****p < 0.0001; ns (not significant) Kruskall–Wallis non-parametric ANOVA. ^a,b,c^*p* < 0.05 multiple comparisons vs. Stage I (^a^), Stage II (^b^) or Stage III (^c^).

**Figure 4:**
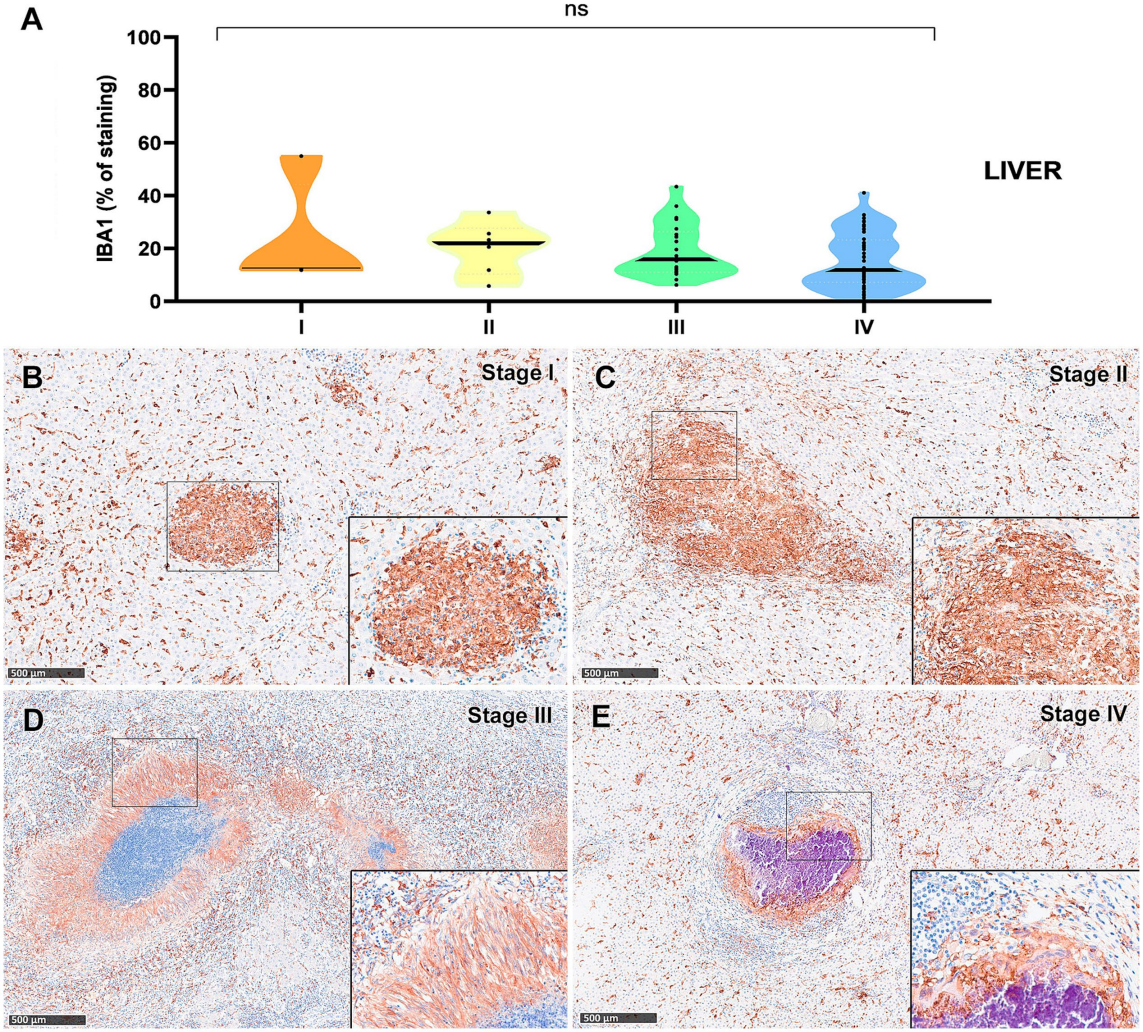
Tuberculous granulomas, alpacas, IBA1 immunohistochemical labelling in stage I, II, III, and IV granulomas in the liver. **(A)** Percentage of IBA1 positive immunostaining in stage I to IV granulomas. **(B)** IBA1 positive immunostaining in stage I granuloma. **(C)** IBA1 expression in stage II granuloma. **(D)** IBA1 positive immunostaining in stage III granuloma surrounding a necrotic core. **(E)** IBA1 positive immunostaining in stage IV granuloma surrounding a necrotic core. Insets show close-up images at higher magnification showing IBA1 immunopositive macrophages **(B,C,E)** and epithelioid macrophages **(D)**. Scale bars = 500 μm (insets = 100 μm). *p < 0.05; ***p < 0.01; ****p < 0.0001; ns (not significant) Kruskall-Wallis non-parametric ANOVA. ^a,b,c^*p* < 0.05 multiple comparisons vs. Stage I (^a^), Stage II (^b^) or Stage III (^c^).

IBA1^+^ cells, identified as macrophages, were the most abundant cell type across all granuloma stages in both lung and liver tissues. However, a progressive decrease in the percentage of IBA1 immunostaining was observed from stage I to stage IV, particularly in the lung (Figures 3A, 4A). In fact, IBA1^+^ cell counts in the lung showed statistically significant differences between early (stages I and II) and advanced granulomas (stages III and IV) (*p* < 0.0001). Notably, the lung granulomas exhibited higher expression of IBA1 + compared to the liver granulomas. The staining was detected on several types of macrophages, including epithelioid and foamy cells and MNGCs, and exhibited a diffuse distribution in all lesions, particularly in early stages (stage I and II) (Figures 3B,C, 4B,C). In advanced stage III and IV granulomas, IBA1-immunopositive staining was also evident in the epithelioid (Figure 4D, inset) and foamy macrophages surrounding the necrotic cores and central mineralization (Figures 3D,E, 4E, insets).

**Figure 5:**
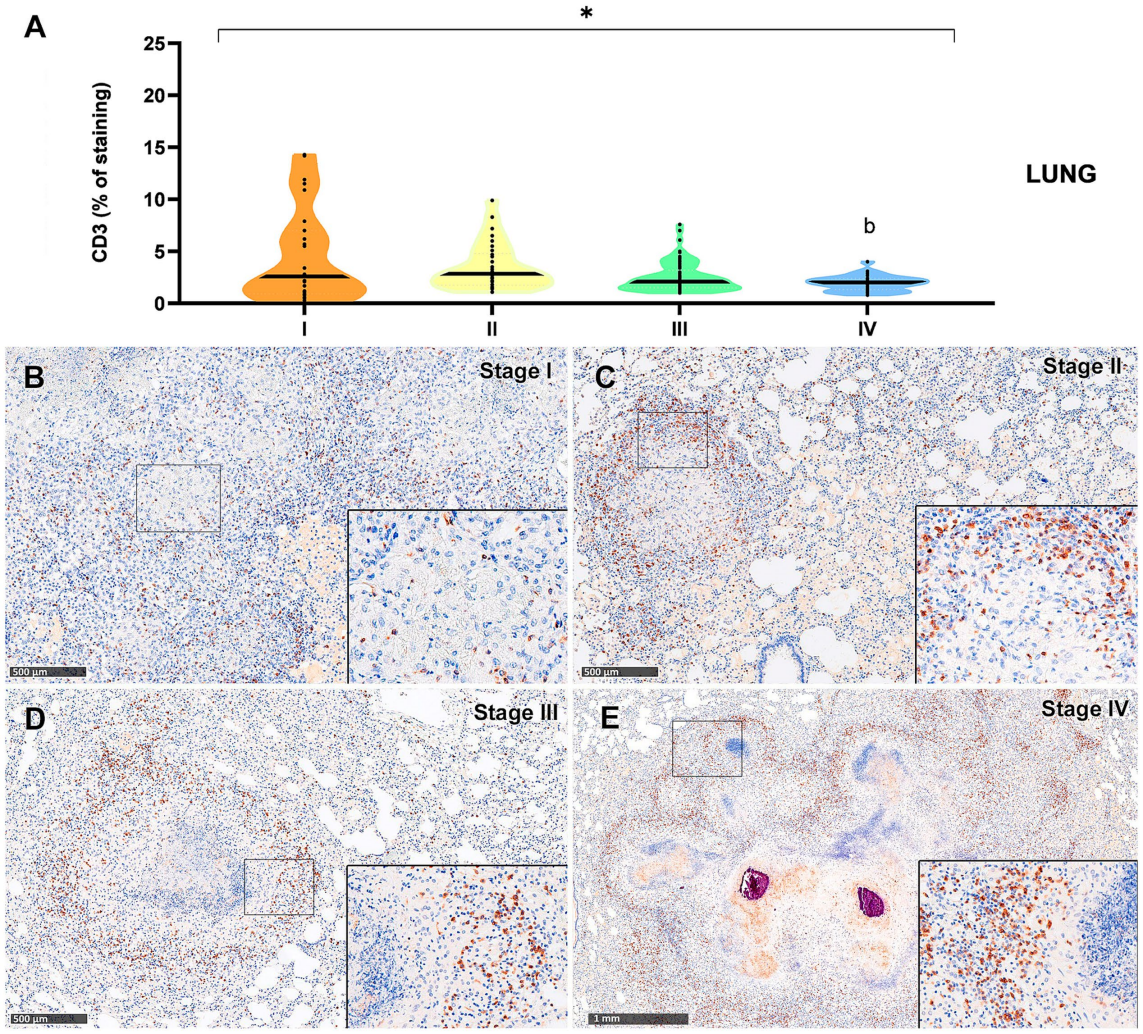
Tuberculous granulomas, alpacas, CD3 positive immunostaining in stage I, II, III and IV granulomas in the lung. **(A)** Percentage of CD3 positive immunostaining in stage I to IV granulomas. **(B)** CD3 positive immunostaining in stage I granuloma. **(C)** CD3 positive immunostaining in stage II granuloma. **(D)** CD3 positive immunostaining in stage III granuloma. **(E)** CD3 positive immunostaining in stage IV granuloma. Insets show close-up images at higher magnification showing CD3 immunopositive T lymphocytes. Scale bars **(B–D)** = 500 μm. Scale bar **(E)** = 1 mm (insets = 100 μm). *p < 0.05; ***p < 0.01; ****p < 0.0001; ns (not significant) Kruskall-Wallis non-parametric ANOVA. ^a,b,c^*p* < 0.05 multiple comparisons vs. Stage I (^a^), Stage II (^b^) or Stage III (^c^).

**Figure 6:**
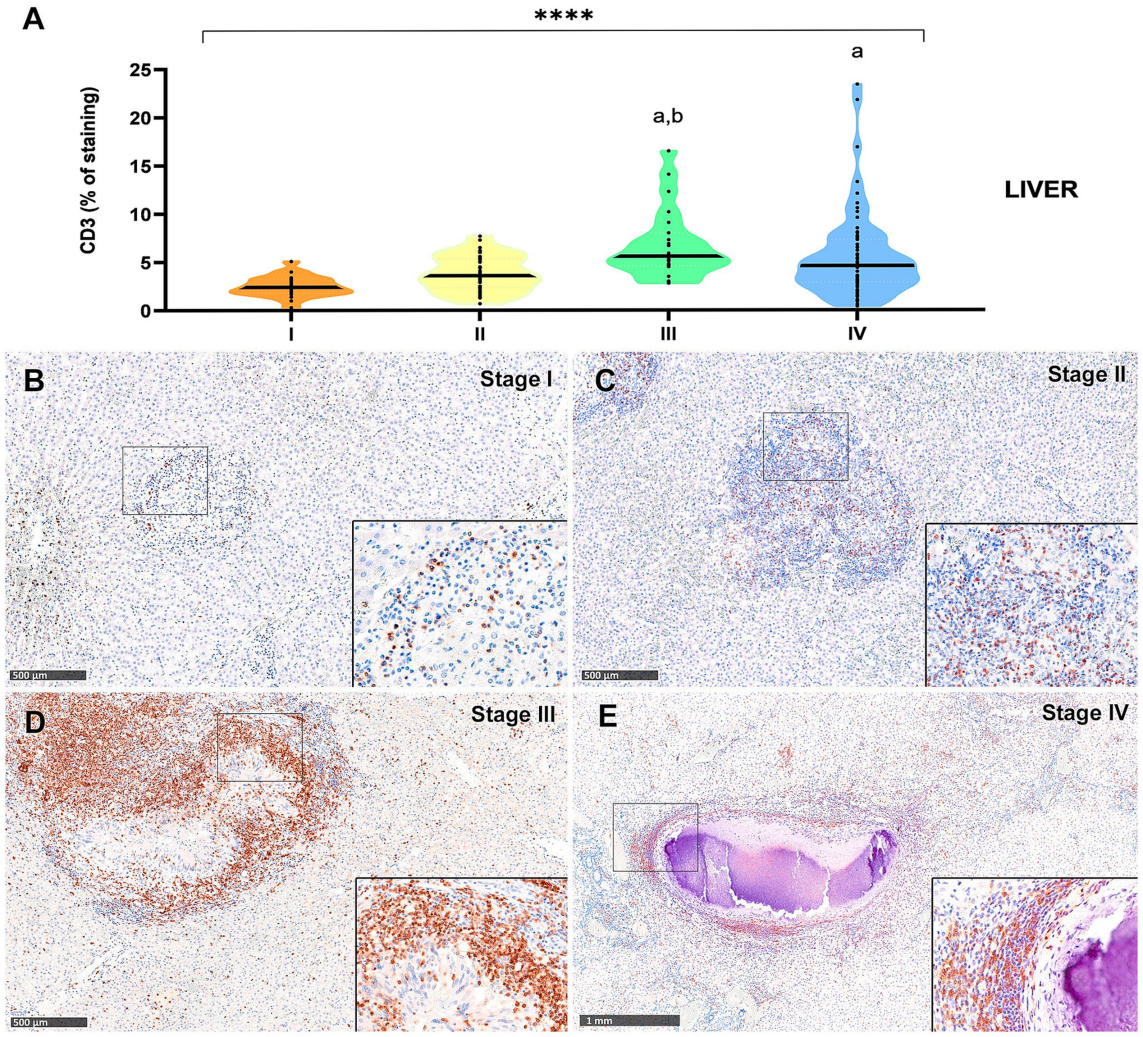
Tuberculous granulomas, alpacas, CD3 positive immunostaining in stage I, II, III, and IV granulomas in the liver. **(A)** Percentage of CD3 positive immunostaining in stage I to IV granulomas. **(B)** CD3 positive immunostaining in stage I granuloma. **(C)** CD3 positive immunostaining in stage II granuloma. **(D)** CD3 positive immunostaining in stage III granuloma. **(E)** CD3 positive immunostaining in stage IV granuloma. Insets show close-up images at higher magnification showing CD3 immunopositive T lymphocytes. Scale bars **(B–D)** = 500 μm. Scale bar **(E)** = 1 mm (insets = 100 μm). *p < 0.05; ***p < 0.01; ****p < 0.0001; ns (not significant) Kruskall-Wallis non-parametric ANOVA. ^a,b,c^*p* < 0.05 multiple comparisons vs. Stage I (^a^), Stage II (^b^) or Stage III (^c^).

Results from CD3-immunopositive staining in lung and liver granulomas are represented in Figures 5A, 6A, respectively. The staining was detected in T lymphocytes, diffusely interspersed within stage I and II granulomas (Figures 5B,C, 6B,C) and located at the outer rims of stage III and IV granulomas from both organs (Figures 5D,E, 6D,E).

In the liver granulomas, CD3^+^ expression increased notably in advanced stages (Figure 6A), with dense aggregates of T lymphocytes observed near the fibrous capsule in stage IV granulomas (Figure 6D). Statistically significant differences were observed between stage III and stage I (*p* < 0.0001), and between stage III and stage II (*p* = 0.003). Stage IV also exhibited significantly higher CD3^+^ cell counts compared to stage I (*p* = 0.0002).

In contrast, CD3^+^ expression in the lung was overall lower, with comparable values between stages II to IV (Figure 5A), although a statistically significant difference was detected between stages II and IV (*p* < 0.01).

**Figure 7:**
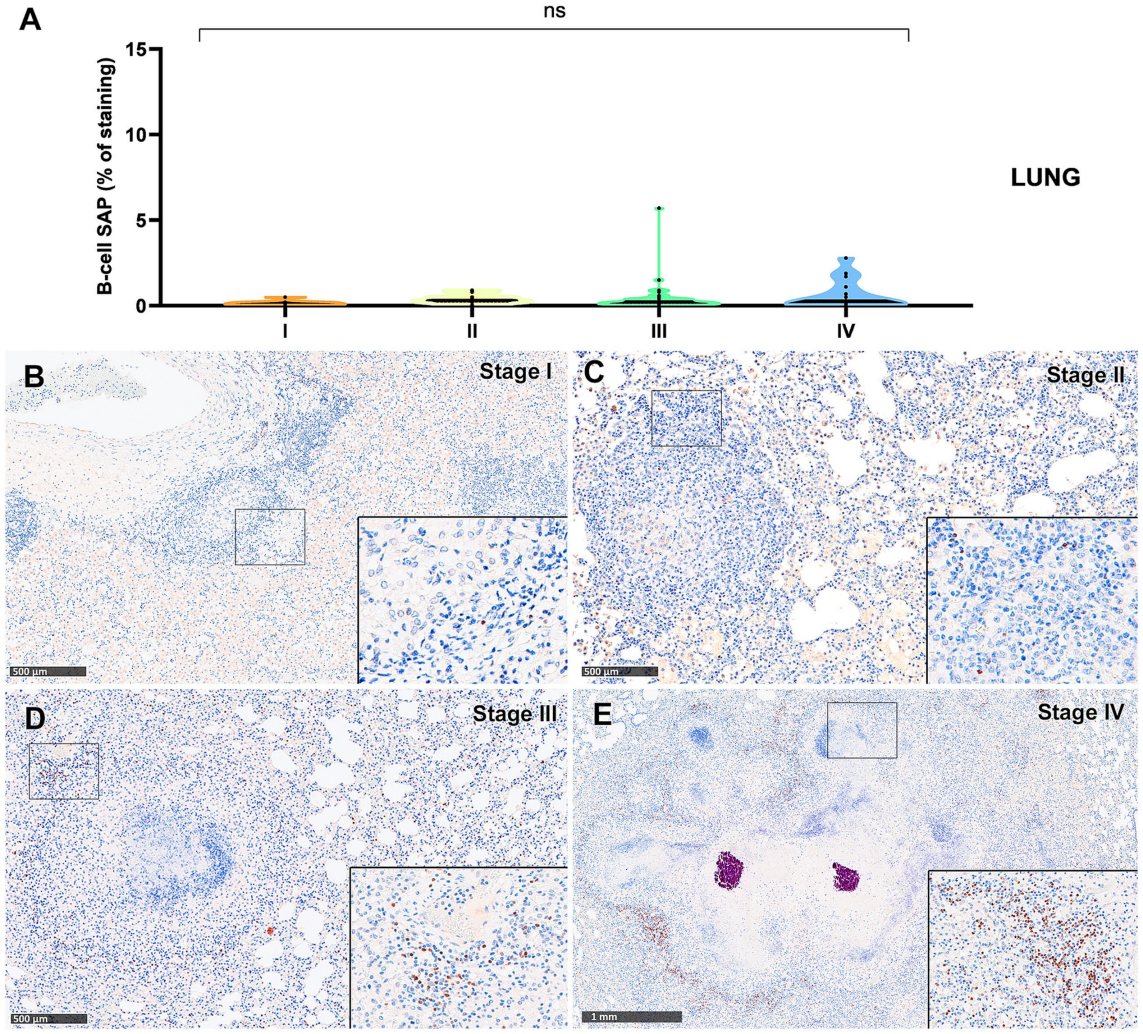
Tuberculous granulomas, alpacas, B-cell Specific Activator Protein^+^ (SAP) immunohistochemical positive immunostaining in stage I, II, III, and IV granulomas in the lung. **(A)** Percentage of B-cell SAP^+^ staining in all stages granulomas. **(B)** B-cell SAP positive immunostaining in stage I granuloma. **(C)** B-cell SAP positive immunostaining in stage II granuloma. **(D)** B-cell SAP positive immunostaining in stage III granuloma. **(E)** B-cell SAP positive immunostaining in stage IV granuloma. Insets show close-up images at higher magnification showing B-cell SAP immunopositive lymphocytes. Scale bars **(B–D)** = 500 μm. Scale bar **(E)** = 1 mm (insets = 100 μm). *P < 0.05; ***p < 0.01; ****p < 0.0001; ns (not significant) Kruskall-Wallis non-parametric ANOVA. ^a,b,c^*P* < 0.05 multiple comparisons vs. Stage I (^a^), Stage II (^b^) or Stage III (^c^).

**Figure 8:**
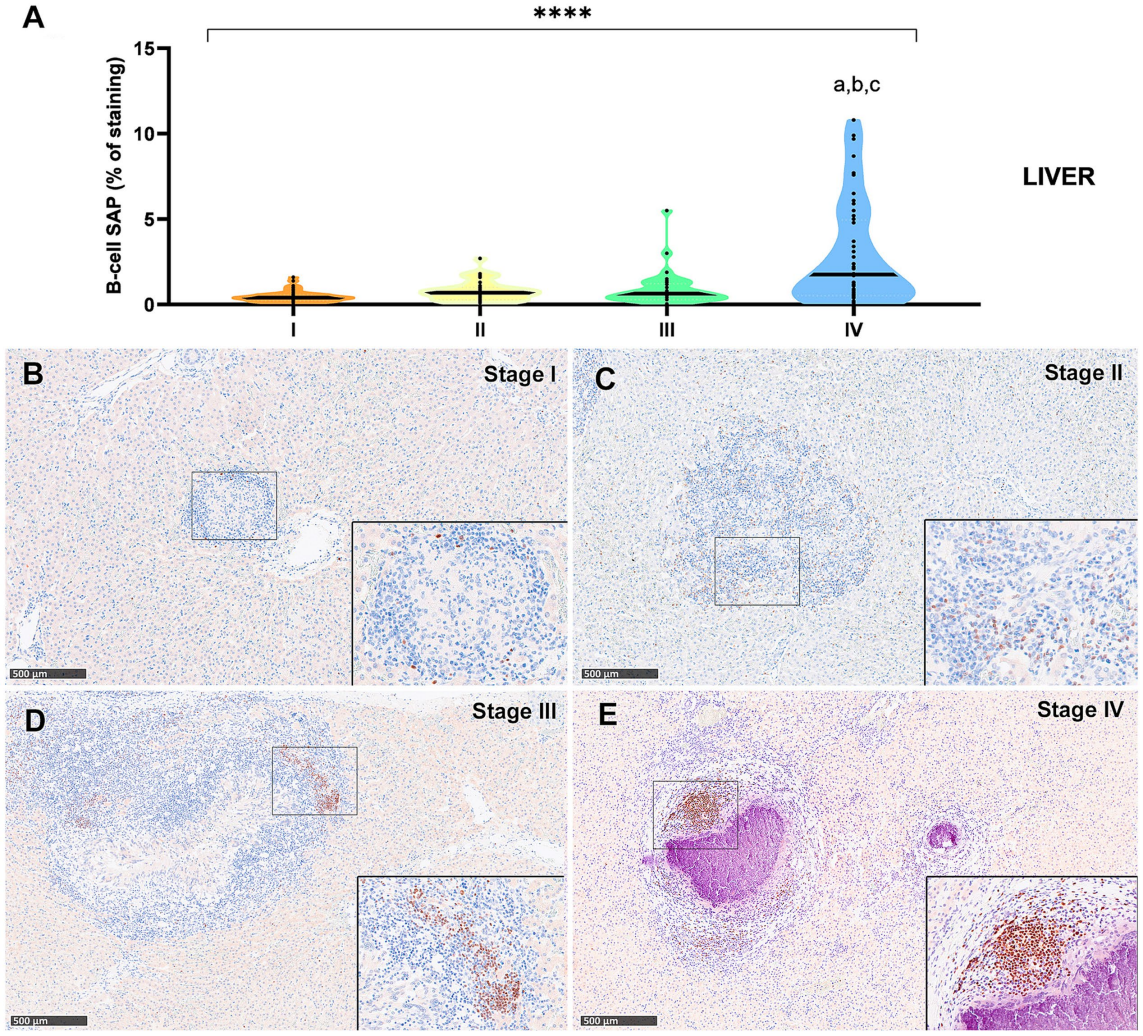
B Tuberculous granulomas, alpacas, B-cell Specific Activator Protein^+^ (SAP) immunohistochemical positive immunostaining in stage I, II, III and IV granulomas in the liver. **(A)** Percentage of B-cell SAP positive immunostaining in all stages granulomas. **(B)** B-cell SAP positive immunostaining in stage I granuloma. **(C)** B-cell SAP positive immunostaining in stage II granuloma. **(D)** B-cell SAP positive immunostaining in stage III granuloma. **(E)** B-cell SAP positive immunostaining in stage IV granuloma manifests as prominent aggregates, displaying characteristic features reminiscent of germinal centers. Insets show close-up images at higher magnification showing B cell SAP immunopositive lymphocytes. Scale bars = 500 μm (insets = 100 μm). * *P* < 0.05; *** *p* < 0.01; ****p < 0.0001; ns (not significant) Kruskall-Wallis non-parametric ANOVA. ^a,b,c^*P* < 0.05 multiple comparisons vs. Stage I (^a^), Stage II (^b^) or Stage III (^c^).

The B-cell marker exhibited a distribution pattern similar to that of T lymphocytes; however, the overall percentage of immunostaining was comparatively lower in both lung (Figure 7A) and liver (Figure 8A) tissues. An increase in SAP^+^ B cells was observed with granuloma progression, reaching the highest percentage in stage IV in both organs (Figures 7A, 8A). Positive B-cell SAP staining was detected in B lymphocytes, diffusely scattered within stage I and II granulomas (Figures 7B,C, 8B,C) and also identified in the periphery of more advanced granulomas (Figures 7D,E, 8D,E). As observed with CD3^+^ cells, dense aggregates of B lymphocytes were noted near the fibrous capsule in stage IV liver granulomas (Figure 8E). In the liver, stage IV granulomas showed significantly higher SAP^+^ B-cell counts compared to all other stages (*p* < 0.05).

**Figure 9:**
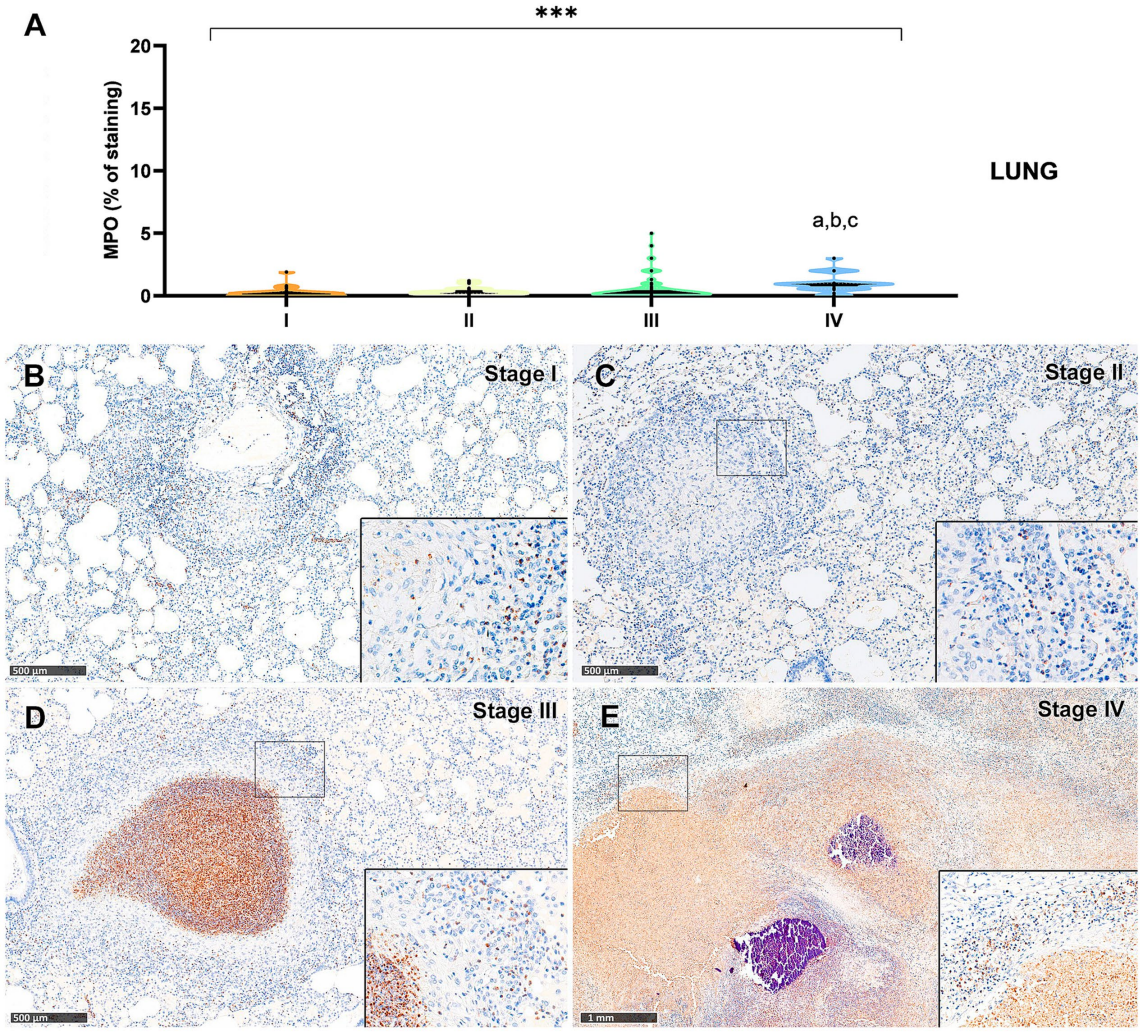
Tuberculous granulomas, alpacas, myeloperoxidase (MPO)^+^ immunostaining in stage I, II, III and IV granulomas in the lung. **(A)** Percentage of MPO positive immunostaining in stage I to IV granulomas. **(B)** MPO expression in stage I granuloma. **(C)** MPO positive immunostaining in stage II granuloma. **(D)** MPO positive immunostaining in stage III granuloma. **(E)** MPO expression in stage IV granuloma. Insets show close-up images at higher magnification showing MPO immunopositive polymorphonuclear cells. Scale bars **(B–D)** = 500 μm. Scale bar **(E)** = 1 mm (insets = 100 μm). *p < 0.05; ***p < 0.01; ****p < 0.0001; ns (not significant) Kruskall-Wallis non-parametric ANOVA. ^a,b,c^*P* < 0.05 multiple comparisons vs. Stage I (^a^), Stage II (^b^) or Stage III (^c^).

**Figure 10:**
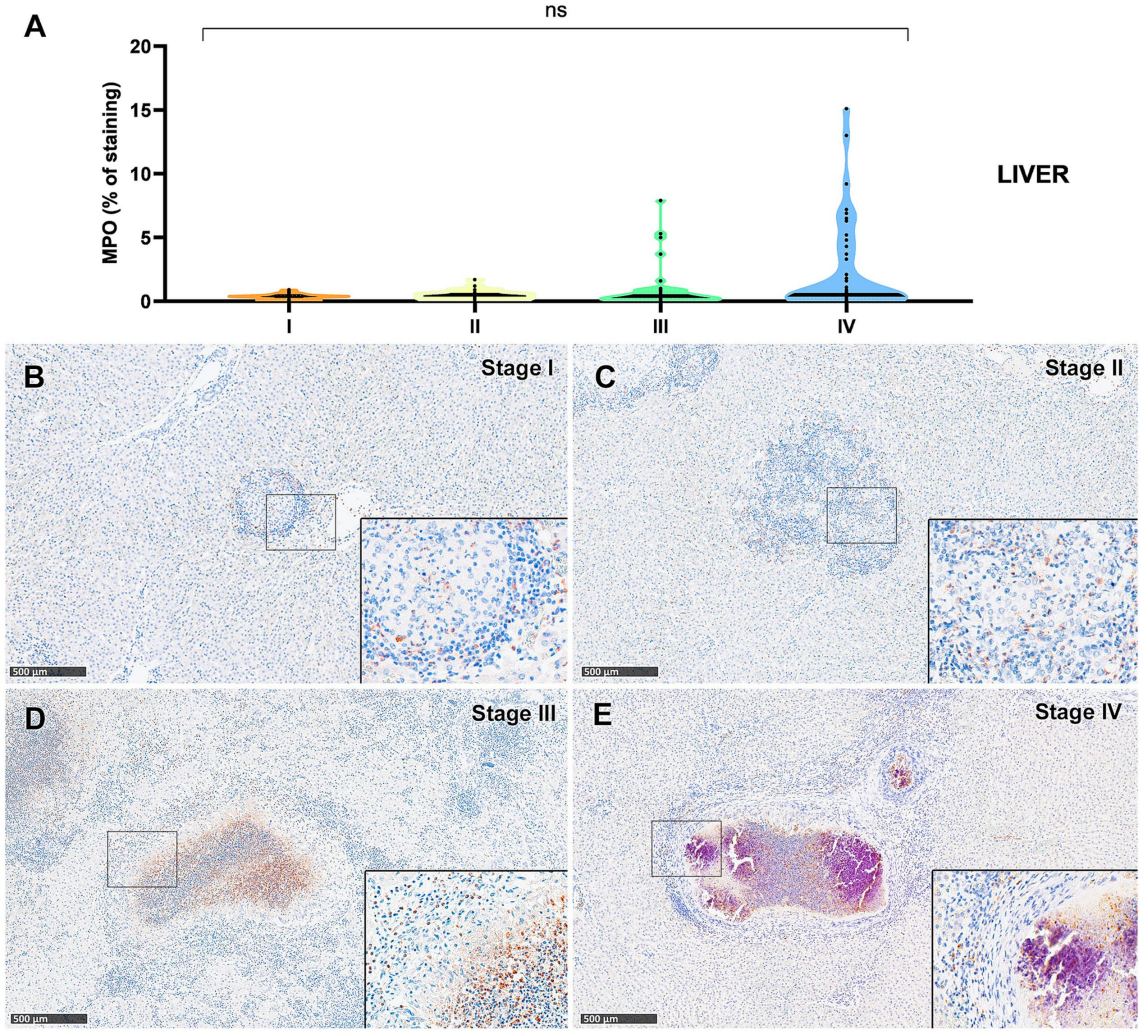
Tuberculous granulomas, alpacas, myeloperoxidase (MPO)^+^ immunostaining in stage I, II, III and IV granulomas in the liver. **(A)** Percentage of MPO positive immunostaining in stage I to IV granulomas in lung **(B)** MPO positive immunostaining in stage I granuloma **(C)** MPO positive immunostainingin stage II granuloma. **(D)** MPO expression in stage III granuloma. **(E)** MPO positive immunostainingin stage IV granuloma. Insets show close-up images at higher magnification showing MPO immunopositive polymorphonuclear cells. Scale bars = 500 μm (insets = 100 μm). *p < 0.05; ***p < 0.01; ****p < 0.0001; ns (not significant) Kruskall-Wallis non-parametric ANOVA. ^a,b,c^*P* < 0.05 multiple comparisons vs. Stage I (^a^), Stage II (^b^) or Stage III (^c^).

Myeloperoxidase (MPO) expression was generally low; however, a progressive increase in the percentage of MPO-positive cells was observed from stage I to stage IV granulomas in both lung (Figure 9A) and liver (Figure 10A). In the lung, stage IV granulomas exhibited significantly higher MPO counts compared to all other stages (*p* < 0.01). The staining was detected in the cytoplasm of interspersed granulocyte-like cells of early stage granulomas (stage I and II) (Figures 9B,C, 10B,C), as well as in the necrotic areas and peripheral inflammatory infiltrates of advanced stage granulomas (stage III and IV) (Figures 9D,E, 10D,E).

## 4 Discussion

Tuberculosis persists as a critical global health concern that claims millions of human lives and imposes considerable economic burdens on livestock worldwide (2, 38). TB in SACs has gained greater importance in Europe in the last decades, since alpacas and llamas are being imported and maintained in growing numbers in several European countries for pet or productive purposes (15, 17, 18, 20). Although TB in ruminants has been extensively studied, the knowledge regarding TB in camelids is still limited. Comprehensive studies on the histopathological aspects of tuberculous granulomas are essential for a better understanding of the disease’s manifestation in these species.

Characterization of TB histological lesions, describing different development stages has been described for several domestic (1, 5) and wildlife (8, 9, 12, 39) species, naturally and experimentally infected, as well as experimental animals (40–42). To the best of the authors’ knowledge, our study describes, for first time, the morphological characteristics associated with development of tuberculous granulomas in tissues from adult alpacas naturally infected with MTBC. We described a well-defined methodology for the systematic classification of these granulomas into distinct developmental stages in this species. Histological assessment of tissue sections revealed different stages of granuloma development within the same organ. A comprehensive categorization was conducted on a total of 175 granulomas in the lungs, 241 in the liver, and 55 in the LNs from alpacas.

The cellular composition of early (stages I-II) and advanced (stages III-IV) granulomas described in the study share similarities with those reported in other species, such as cattle (43–45) and guinea pigs (42). These encompass initial lesions characterized by small clusters of immune cells, predominantly activated macrophages, accompanied by varying quantities lymphocytes and neutrophils, progressing to extensive necrotic areas with degenerated neutrophils and mineralization surrounded by a rim of activated macrophages and clusters of T and B cells. Additionally, liver granulomas showed a peripheral thick connective capsule in stage IV granuloma, a feature described in other animal species such as non-human primates (NHP) (40, 46) and cattle (45). In the studied organs, III and IV stage granulomas were the most frequent in all the tissues evaluated, indicative of the chronicity and severity of the process, as reported in cattle by other authors (47).

Additionally, the presence of MNGCs in TB granulomas from all developmental stages is very rare, in contrast to other domestic and wildlife species showing abundant numbers of MNGCs, like cattle or deer (6, 7, 43). However, other animal species show none of very few MNGCs within TB granulomas, like small rodents (41) or non-human primates (40, 41, 46).

Interestingly, the liver emerged as the most affected organ in the evaluated animals, suggesting the oral route as being quite relevant in this species. This finding aligns with observations in orally infected calves, where abdominal organs were the most affected (48). While mycobacteria typically reach the liver through haematogenous dissemination, generally from the lungs, resulting in a miliary form, local spread from the gastrointestinal tract via portal vein has also reported (49).

A high percentage of granulomas showing AFBs with the ZN staining were observed in the lung (92.00%, 161/175) and LNs (89.1%, 49/55), contrasting with findings in other species with a paucibacillary lesion pattern, such as cattle (6, 50), buffalo (6) and wild boar (6, 7). The presence of AFBs was observed in all the granuloma stages, with an increase in their numbers observed simultaneously with lesion development, as described in other animal species (42, 43). The diffuse pattern observed in the lung and associated lymph nodes, characterized by extensive necrotic and unencapsulated lesions with a high presence of AFBs, suggests a breakdown in lesion control by the host immune response, as previously described in this species (15, 18). This indicates that alpacas may act as a potential source of pathogenic mycobacterial excretion, posing a transmission risk for domestic animals, wildlife and even humans. Conversely, 56.84% (137/241) of hepatic granulomas, characterized by the presence of a thick connective tissue capsule in stage IV lesions, exhibited no detectable AFBs. This observation aligns with the significantly enhanced capacity to control mycobacterial growth in the liver compared to the lung, as demonstrated by previous studies (51, 52). Notably, the liver sinusoids can serve as a barrier to limited mycobacterial infection (53). Additionally, Seiler et al. in 2001 (54) proposed that the liver’s distinctive resistance to mycobacteria results from the exclusive infection of “professional” phagocytes within this organ, a restriction likely reinforcing host defense against mycobacterial proliferation. In contrast, within the lung, mycobacteria can infect various host cells, including non-phagocytic ones. The inherent inability of non-phagocytic cells to eliminate and process mycobacteria may potentially facilitate mycobacterial persistence.

Additionally, this study describes the distribution of the main cells involved in alpacas´ granuloma development and demonstrates the cross-reactivity of a set of immunohistochemical markers for distinct alpaca cell subsets (IBA1, MPO, CD3, and B-cell-SAP). A representative number of granulomas from each histological stage were analysed in both lung and liver tissues to ensure adequate characterization of cell populations throughout granuloma progression. Macrophages, identified by the IBA1 marker, constituted the most prevalent cell population detected, and were the unique cell subset exhibiting a statistically significant decline in positive immunostaining as granulomas progressed in lung. A similar reduction was also observed in the liver granulomas, although it did not reach statistical significance. These findings are consistent with those that showed a decreasing percentage of positivity by stage in both the lungs and liver, in accordance with findings reported by Garcia-Jimenez et al. in 2013 (9) in naturally infected wild boar. These observations underscore the crucial role of these phagocytes in the initial stages of granuloma formation. T lymphocytes, detected by the CD3 marker, underwent changes within different granuloma stages. Initially showing diffusely scattered staining within stage I and II granulomas, and located in higher numbers, at the periphery of stages III and IV, as described previously in cattle (45), guinea pig (42), and wild boar (9). These changes reflect the dynamic process of granuloma development and signify the local organization of various lymphocyte subsets in response to mycobacteria infection (9, 40, 55). The increase in CD3^+^ expression observed in advanced hepatic granulomas, with statistically significant differences compared to earlier stages, may suggest an enhanced and spatially organized adaptive response, potentially contributing to the containment of necrotic lesions in this organ, contrasting with that observed in the lung. B-cell-SAP labelling followed a similar distribution pattern than CD3 marker, and showed an increase on the percentage of positivity by stage in both organs, primarily in the lungs. In alpacas´ granulomas, B lymphocytes were observed forming peripheral lymphoid follicle-like structures in the advanced granulomas, a feature described by other authors in mice (56), cattle (57), NHPs (46) and humans (55). Importantly, the significant increase of B-cell counts in stage IV liver granulomas highlights their potential role in tertiary lymphoid structure formation, a phenomenon linked to chronic antigenic stimulation and prolonged inflammation in mycobacterial infections. Neutrophils, identified by MPO, were the least abundant cells but increased notably in necrotic areas and lesion peripheries of advanced granulomas. This distribution suggests their involvement in both tissue destruction and the modulation of the local immune response during granuloma progression, in line with observations reported in guinea pigs (42).

## 5 Conclusion

In conclusion, this study characterizes, for the first time, the immunopathology of granuloma development in naturally infected alpacas with *M. bovis*. We present a well-defined methodology for their classification into stages, including a detailed description of the involved cellular populations. The elevated incidence of tuberculous lesions observed in the liver implies a high frequency of generalized tuberculosis in this particular species. Additionally, the high prevalence of AFBs reported in the lungs and LNs implies potential transmission risks.

This research not only addresses knowledge gaps about pathological features of tuberculous granulomas in camelids, but also underscores alpacas as potential sources of mycobacterial excretion for domestic animals, wildlife and humans.

## Data Availability

The raw data supporting the conclusions of this article will be made available by the authors, without undue reservation.
